# AG490 suppresses EPO-mediated activation of JAK2-STAT but enhances blood flow recovery in rats with critical limb ischemia

**DOI:** 10.1186/s12950-016-0126-3

**Published:** 2016-06-04

**Authors:** Han-Tan Chai, Hon-Kan Yip, Cheuk-Kwan Sun, Shu-Yuan Hsu, Steve Leu

**Affiliations:** Division of Cardiology, Department of Internal Medicine, Kaohsiung Chang Gung Memorial Hospital and Chang Gung University College of Medicine, Kaohsiung, Taiwan; Institute for Translational Research in Biomedicine, Kaohsiung Chang Gung Memorial Hospital, Kaohsiung, Taiwan; Center for Shockwave Medicine and Tissue Engineering, Kaohsiung Chang Gung Memorial Hospital, Kaohsiung, Taiwan; Department of Medical Research, China Medical University Hospital, China Medical University, Taichung, Taiwan; Department of Nursing, Asia University, Taichung, Taiwan; Department of Emergency Medicine, E-Da Hospital, I-Shou University, Kaohsiung, Taiwan; Department of Anatomy, Graduate Institute of Biomedical Sciences, ,College of Medicine, Chang Gung University, Taoyuan, Taiwan

**Keywords:** Erythropoietin, AG490, JAK2, Critical limb ischemia, Apoptosis

## Abstract

**Background:**

Erythropoietin (EPO) has been demonstrated to enhance recovery in ischemic organs through enhancing angiogenesis. In this study, we used an experimental critical limb ischemia (CLI) rat model to reveal the underlying mechanisms and directly examine the benefits of the anti-apoptotic capacity of EPO in the acute phase of limb ischemia and following blood flow recovery.

**Methods:**

To determine the role of the JAK2/STAT pathway in EPO-enhanced recovery after CLI, male Sprague-Dawley rats (*n* = 8 for each group) were divided into group 1 (normal control), group 2 (CLI treated with normal saline), group 3 (CLI treated with EPO), group 4 (CLI treated with AG490, a JAK2 inhibitor), and group 5 (CLI treated with EPO and AG490). Animals were sacrificed either at day 1 or day 14 and biochemical and histopathological examination of ischemic quadriceps were conducted.

**Results:**

At day 1, EPO administration reduced expression levels of apoptotic indices and activated the JAK2/STAT pathway; this activation was inhibited by additional AG490 treatment. Furthermore, the decrease in the size of the infarcted area, as well as activation of ERK1/2 and JNK showed similar regulatory trends with EPO or AG490 treatment. Of Interest, EPO and AG490 in combination showed a synergistic effect, increasing expression levels of antioxidants (GR, GPx, NQO-1) and decreasing transcriptional levels of pro-inflammatory factors (TNF-α, NF-kB). At day 14, laser Doppler analysis showed that the blood flow recovery was enhanced by EPO, AG490, or combined treatment.

**Conclusion:**

Although inhibition of the JAK2/STAT pathways reduces the anti-apoptotic effects of EPO in the early phase of CLI, the benefits of AG490 in anti-inflammation and anti-oxidation still play a positive role in enhancing blood flow recovery after CLI.

## Background

Peripheral arterial disease (PAD) is caused by chronic inflammatory processes associated with atherosclerosis [1]. Critical limb ischemia (CLI), which results in significant blood flow reduction in feet and hands, is the most severe form of PAD [2]. Although endovascular intervention and open surgical techniques are widely used treatments for CLI, amputation remains the final option for a certain subset of patients [3–5]. Under general medical care conditions, one year after diagnosis of CLI, half of patients are dead or alive with amputations, while only quarter of patients see symptoms resolve [2]. So far, there is no satisfying pharmacologic therapy to efficiently reverse arterial occlusive lesions, or the subsequent impaired perfusion in ischemic limbs of patients [6]. The purpose of pharmacologic treatment for CLI includes risk factor modification and efforts to improve blood flow [7, 8]. However, only patients with mild to moderate intermittent claudication are advised to undergo pharmacologic therapy [7]. Therefore, alternate treatment approaches are urgently needed for CLI.

Erythropoietin (EPO), a 165 kDa secreted glycoprotein, was first characterized as a hematopoietic factor and has been widely used for the clinical treatment of anemia [9–11]. EPO not only promotes the proliferation and differentiation of erythroid precursors, but also plays an important role as an anti-apoptotic factor for hematopoietic cells [12]. In general, the expression level of erythropoietin is upregulated under hypoxic conditions and mediated by a transcription factor, hypoxia inducible factor-1 (HIF-1) [13, 14]. EPO is mainly produced by cells of the peritubular capillary endothelium of the kidney [15], while EPO receptors (EPOR) are widely expressed in various tissues, including brain, retina, heart, kidney, smooth muscle, myocardium, and endothelium [15]. The EPO-mediated protective responses in anti-apoptosis are also found in non-hematopoietic cells, e.g., renal tubular cells [16], neurons [17], retina cells [18], cardiomyocytes [19], and endothelial cells [17]. Recent studies also demonstrated that EPO plays multiple functional roles in anti-inflammation [15, 20], angiogenesis [21, 22], and in endothelial response to increasing nitric oxide production [23]. The therapeutic efficacy of EPO in amelioration of organ ischemic injury or ischemia-reperfusion injury has been evaluated through experimental animal models as well as clinical applications [24–28].

EPO is activated through its binding to the EPO receptor (EPOR), which is composed of two identical subunits [29, 30]. After binding, the receptor is dimerized and Janus kinase-2 (JAK2) is then recruited to the receptor complex [29, 30]. After binding of EPO and EPOR, several substrates of JAK2, including transcription factor signal transducer and activator of transcription (STAT) are recruited to the docking site of EPOR [29, 30]. STATs are phosphorylated by JAK kinases, leading to dimerization and subsequent translocation to the nucleus [29, 30]. After nuclear translocation, STATs bind to promoters of several genes involved in anti-apoptosis, including Bcl-xL, Bcl-2 and c-Myc. However, although the activation of JAK2/STATs plays an anti-apoptotic role in organ injury, this activated signaling is also involved in upregulation of pro-inflammatory cytokine generation [31–33]. Inhibition of JAK2 activity through its inhibitors (i.e., AG490) has been applied as an approach to treat ischemia-reperfusion injury and autoimmune arthritis in animal models [34, 35]. Hence, revealing the underlying mechanism of EPO-mediated cellular response is important for the selection and adjustment of clinical application of EPO in different types of organ injuries.

Although EPO has been demonstrated to have therapeutic efficacy in treating critical limb ischemia in experimental animal models [22], the underlying mechanisms are still not completely clear. In the present study, we applied rat CLI models with a JAK2 inhibitor to determine the whether the JAK2/STAT pathway is essential for EPO-mediated blood flow recovery after CLI. In addition, whether the anti-inflammatory role of AG490 contributes in injury recovery from CLI was also examined.

## Methods

### Animal model of critical limb ischemia

Pathogen-free, adult male Sprague-Dawley (SD) rats (*n* = 60) weighing 320-350 g (Charles River Technology, BioLASCO Taiwan Co. Ltd., Taiwan) were used in this study (*n* = 16 for each group): Group 1, normal control; Group 2, critical limb ischemia (CLI) with normal saline; Group 3, CLI with EPO; Group 4, CLI with AG490; Group 5, CLI with EPO and AG490. EPO was injected intramuscularly (1000 IU/kg), while AG490 was injected intraperitoneally (3 mg/kg). EPO and AG490 were administrated at 30 min, 24 h, and 48 h after induction of CLI. Animals were sacrificed at either day 1 or day 14 (8 animals from each group were sacrificed at each time-point). For induction of critical limb ischemia by ligation of the femoral artery, rats were placed in a supine position on a warming pad at 37 °C with the left hind limbs shaved. Under sterile conditions, the left femoral artery, small arterioles and circumferential femoral artery were exposed and ligated over their proximal and distal portions before removal. To avoid the presence of collateral circulation, the branches were removed together. After sacrifice, the left quadricep muscles were collected for individual study.

### Measurement of blood flow with laser doppler

Rats were anesthetized by inhalation of 2.0 % isoflurane prior to CLI induction and on days 2 and 14 after CLI induction prior to being sacrificed (*n* = 8 for each group). The rats were placed in a supine position on a warming pad at 37 °C. After being shaved over bilateral hind limbs and inguinal areas, blood flow was surveyed by a Laser Doppler scanner (moorLDLS, Moor, UK). The ratio of blood flow in the left hind limb (ischemic) to that in the right side (normal) was applied to determine the blood flow recovery after CLI.

### Quantitative reverse transcription-polymerase chain reaction

Quantitative mRNA levels were determined using real-time reverse transcription-polymerase chain reaction (RT-PCR) with the Applied Biosystems 7900 HT Sequence Detection System (Applied Biosystems) and TaqMan Gene Expression Assay as previously described [36].

### Western blot analysis

Equal amounts (10-30 μg) of protein extracts from ischemic quadriceps of the animals (*n* = 6 for each group) were loaded and separated by SDS-PAGE using 7 or 12 % acrylamide gradients. Proteins were then transferred to nitrocellulose membranes. The membranes were incubated with monoclonal antibodies against JAK2 (1:1000, Abcam), phosphorylated JAK2 (1:1000, Abcam), STAT1 (1: 1000, Cell Signaling), phosphorylated STAT1 (1: 1000, Cell Signaling), STAT3 (1:500, Cell Signaling), phosphorylated STAT3 (1:1000, Cell Signaling), STAT5 (1:1000, Abcam), phosphorylated STAT5 (1:500, Abcam), Akt (1:1000, Cell Signaling), phosphorylated Akt (1:2000, Cell Signaling), JNK (1:500, Sigma), phosphorylated JNK (1:1000, Abcam), ERK1/2 (1:1000, Cell Signaling), and phosphorylated ERK1/2 (1:2000). Signals were detected with HRP-conjugated goat anti-mouse or goat anti-rabbit IgG. Immunoreactive bands were visualized by enhanced chemiluminescence (ECL; Amersham Biosciences) which was then exposed to Biomax L film (Kodak). For quantification, ECL signals were digitized using Labwork software (UVP).

### Histopathological and immunostaining

The immunofluorescence (IF) staining and immunohistochemical (IHC) staining were performed as previously described [37]. In brief, fixed cryosections (10 μm) of quadriceps were incubated with antibodies against CD31 (1:200, Abcam), EPOR (1:500, Abcam), or α-SMA (1:400, Millipore) at 4 °C overnight, followed by incubation with fluorescence or HRP-conjugated secondary antibodies. For quantification, ten randomly selected HPFs (high power fields, 200×) were analyzed in each section. The mean number per HPF for each animal was then determined by summation of all numbers divided by 30.

### Statistical analysis

Data was expressed as mean values (mean ± SD). The significance of differences between two groups was evaluated with *t*-test. The significance of differences among groups was evaluated using one-way ANOVA, followed by Bonferroni multiple comparison post hoc test. Statistical analysis was performed using Prism 5 statistical software (GraphPad Software, La Jolla, CA, USA). A probability value of less than 0.05 was considered statistically significant.

## Results

### AG490 administration inhibits phosphorylation of STATs in quadriceps

**Fig. 1:**
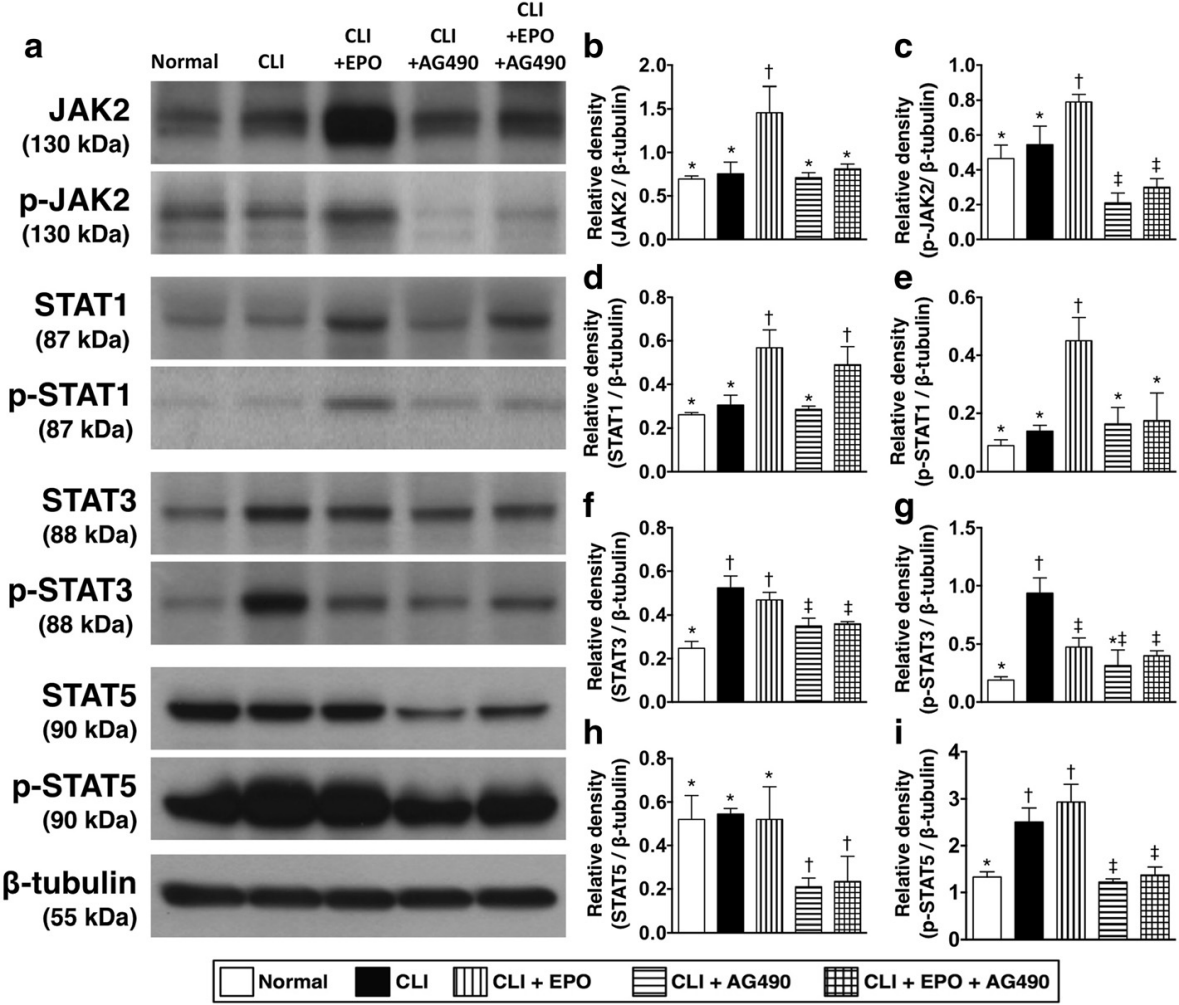
EPO regulates the expression and phosphorylation levels of JAK/STATs in quadriceps after critical limb ischemia. **a** Twenty-four hours after CLI, total protein was extracted from quadriceps and Western blots were performed with antibodies against JAK2, phospho-JAK2, STAT5, phospho-STAT5, STAT1, phospho-STAT1, STAT3, and phospho-STAT3. **b** and **c** The expression levels of total and phosphorylated JAK2. **d** and **e** The expression levels of total and phosphorylated STAT1. **f** and **g** The expression levels of total and phosphorylated STAT3. **h** and **i** The expression levels of total and phosphorylated STAT5. Statistical analysis used one-way ANOVA followed by Bonferroni multiple comparison post hoc test (*n* = 8 for each group). Symbols (*, †, ‡,) indicate significance at p value less than 0.05. EPO, Erythropoietin; JAK, Janus kinase; STAT, Signal Transducer and Activator of Transcription

To clarify the activation of the JAK2 pathway in EPO-mediated enhanced recovery from limb ischemia, AG490, a well-known JAK2 inhibitor, was applied to rats that underwent femoral artery ligation. Apoptosis occurs in the acute phase of ischemia. Since EPO has been reported to function in the inhibition of apoptosis, the quadriceps were isolated to examine the activation of JAK2 and its downstream STATs in the ischemic area (Fig. 1a). Results showed that both total and phosphorylated JAK2 was significantly higher in CLI rats with EPO administration (Fig. 1b and c). The increased JAK2 phosphorylation in quadriceps of EPO-treated CLI rats was blocked by AG490 (Fig. 1c). To further confirm the activation of the JAK2 pathway in CLI rats with EPO treatment, the expression levels and phosphorylation status of STAT family proteins in ischemic quadriceps were then examined (Fig. 1a). Along with the activation of EPO, the expression levels of phosphorylated STAT1 (Fig. 1e) and STAT5 (Fig. 1i) were increased in quadriceps of CLI rats with EPO treatment. The EPO-mediated activation of the STATs was also abolished with AG490. Of interest, the total expression and phosphorylated levels of STAT3 were significantly increased by induction of CLI. These increases of STAT3 levels were not further enhanced by EPO treatment, but were abolished by AG490 (Fig. 1f and g).

### EPO administration showed an anti-apoptotic activity in ischemic quadriceps

**Fig. 2:**
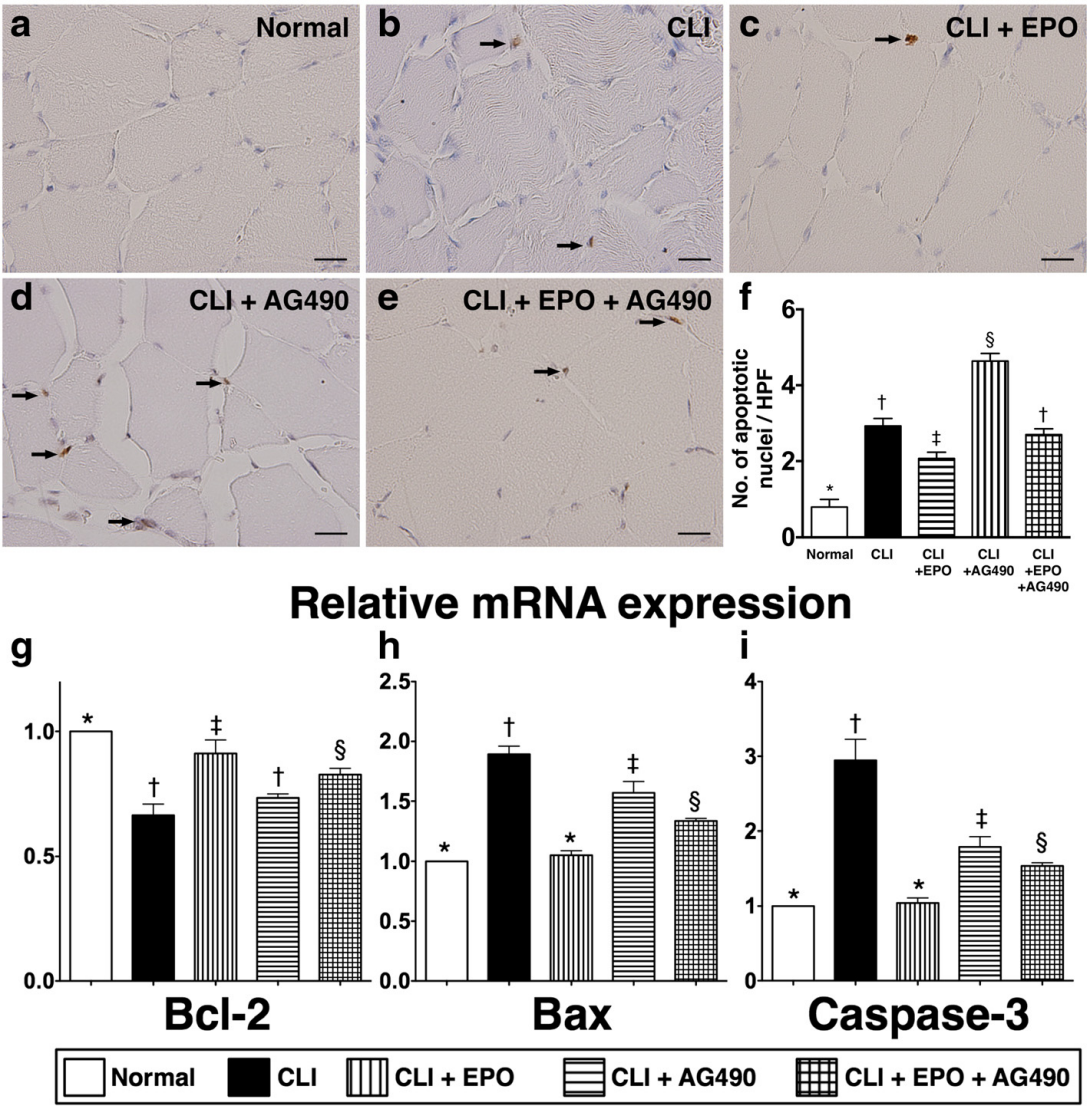
EPO administration reduces the number of apoptotic nuclei and regulates transcriptional levels of apoptosis indices. Twenty-four hours after CLI, quadriceps from ischemic limbs were isolated for cryosections and total RNA extraction. Cryosections were used for terminal deoxynucleotidyl transferase dUTP nick end labeling (TUNEL) assay to detect apoptotic nuclei, and total RNA was used for real-time reverse transcription polymerase chain reaction (RT-qPCR) with specific primers to determine the mRNA expression levels of Bcl-2, Bax, and caspase-3. **a**-**e** The apoptotic nuclei were identified with TUNEL assay and indicated with black arrows. **f** The quantitation of the number the apoptotic nuclei. **g** The mRNA expression level of Bcl-2, an anti-apoptotic index. **h** and **i** The mRNA expression levels of Bax and caspase-3, two apoptotic indices. Statistical analysis used one-way ANOVA followed by Bonferroni multiple comparison post hoc test (*n* = 8 for each group). Symbols (*, †, ‡, §) indicate significance at p value less than 0.05

Although EPO has been found to have an anti-apoptotic function in vitro in cultured endothelial cells [38], its physiological anti-apoptotic role has not been confirmed in vivo. Hence, TUNEL assays were performed in situ to detect the apoptotic nuclei in ischemic quadriceps (Fig. 2a-f). Results showed that the number of apoptotic nuclei was increased after CLI induction (Fig. 2b) and this increase was reversed with EPO treatment (Fig. 2c). The administration of AG490 further increased the number of apoptotic nuclei in CLI rats (Fig. 2d). Moreover, reduction of the number of apoptotic nuclei in quadriceps of CLI rats by EPO was blocked by additional AG490 treatment (Fig. 2e).

Quantitated real-time reverse transcription polymerase chain reaction (RT-qPCR) was also performed to confirm the results of TUNEL assay (Fig. 2g-i). RT-qPCR examination of transcripts of Bax (Fig. 2h) and caspase-3 (Fig. 2i), two pro-apoptotic indices showed similar regulatory trends to those observed in the TUNEL assay. In contrast, mRNA expression levels of Bcl-2, an anti-apoptotic factor, were decreased after CLI induction and reverted with EPO treatment (Fig. 2g). In addition, the blocking of EPO-mediated cellular protective function against apoptosis in ischemic quadriceps by AG490 was confirmed by RT-qPCR (Fig. 2g-i).

### Infarct area in ischemic quadriceps is reduced by EPO treatment

**Fig. 3:**
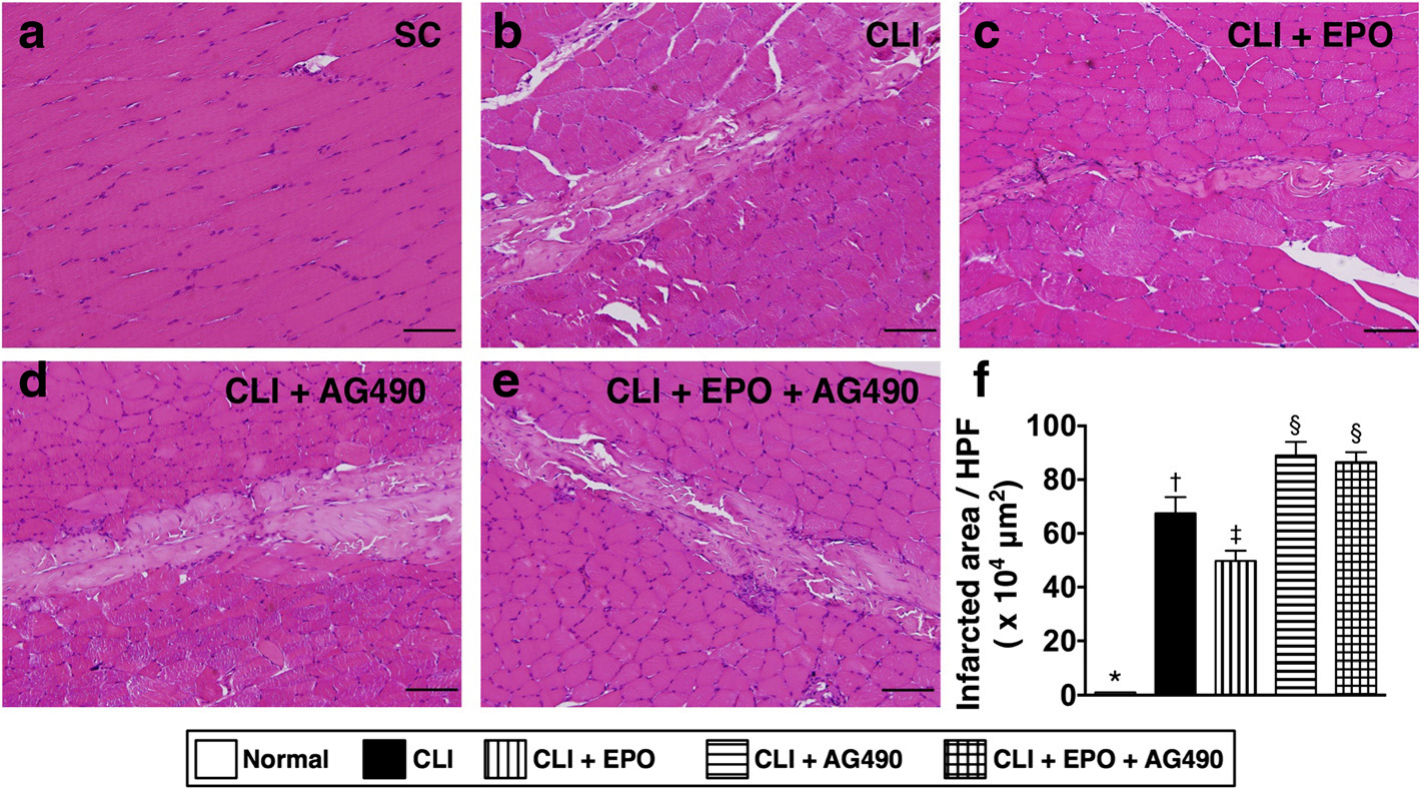
EPO reduces the infarcted area in ischemic quadriceps. The infarcted area of the quadriceps was determined using Hematoxylin and Eosin (H&E) staining. **a** Quadricep section from normal rats. **b** Quadricep section from rats with critical limb ischemia (CLI). **c** Quadricep section from CLI rats treated with EPO. **d** Quadricep section from CLI rats treated with AG490. **e** Quadricep section from CLI rats treated with EPO and AG490. **f** Quantitative evaluation of the infarct areas in quadriceps. Statistical analysis used one-way ANOVA followed by Bonferroni multiple comparison post hoc test (*n* = 8 for each group). Symbols (*, †, ‡, §) indicate significance at p value less than 0.05

After CLI induction, the infarction of skeletal muscles is usually accompanied by with loss of blood flow and cellular apoptosis. Following the examination of apoptotic events at the tissue and molecular levels, histopathological examination was performed to determine the infarcted area in the ischemic quadriceps (Fig. 3). Quadriceps from rats were isolated and used for preparing cryo-section followed by Hematoxylin and Eosin (H&E) staining. Infarcted areas in the quadriceps of all groups were measured and calculated. The results showed that the infarct areas increased after CLI induction and that this increase was reverted with EPO treatment (Fig. 3b and c). AG490-only treatment increased the infarct area in the quadriceps of CLI rats (Fig. 3d), and reduction of the infarcted area in the ischemic quadriceps by EPO was inhibited by additional treatment with AG490 (Fig. 3e).

### EPO and AG490 contribute to anti-inflammation and anti-oxidation in the ischemic quadriceps

**Fig. 4:**
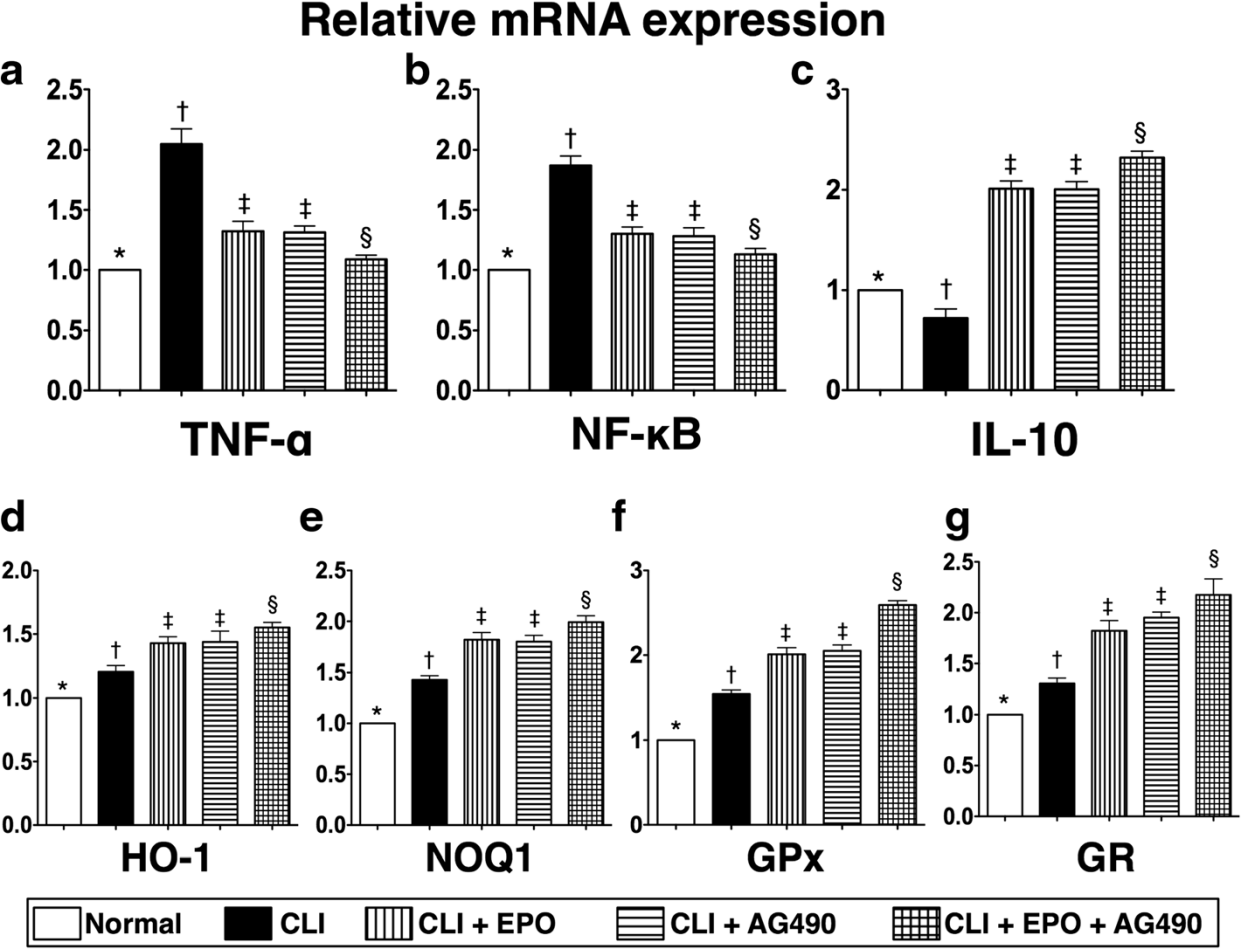
EPO and AG490 administrations reduce the mRNA expression levels of inflammatory factors and increase the expression levels of antioxidants. Twenty-four hours after CLI, total RNA extracted from quadriceps was used for real-time reverse transcription polymerase chain reaction (RT-qPCR) to determine the mRNA expression levels of TNF-α, NF-kB, IL-10, HO-1, NQO-1, GPx and GR. **a** The mRNA expression levels of TNF-α. **b** The mRNA expression levels of NF-kB. **c** The mRNA expression levels of IL-10. **d**-**g** The mRNA expression levels of HO-1, NQO-1, GPx, and GR, four antioxidants. Statistical analysis used one-way ANOVA followed by Bonferroni multiple comparison post hoc test (*n* = 8 for each group). Symbols (*, †, ‡, §) indicate significance at p value less than 0.05

After ischemic injury, oxidative stress and inflammation usually impair tissue regeneration and micro-circulation reconstruction. Hence, the regulation of expression levels of inflammatory and anti-oxidative factors by EPO and AG490 in ischemic quadriceps was examined. Twenty-four hours after CLI induction, the transcriptional levels of inflammatory and anti-inflammatory factors were examined using RT-qPCR (Fig. 4). Results showed that gene expression levels of tumor necrosis factor (TNF)-α and nuclear factor kappa-light-chain-enhancer of activated B cells (NF-kB), two inflammatory factors, were increased after CLI induction, while both EPO and AG490 reduced their expression levels in ischemic quadriceps (Fig. 4a and b). It is worth noting that, instead of inhibition, combined AG490 and EPO treatment showed a synergistic effect in reducing the expression levels of inflammatory cytokines. The transcriptional levels of interleukin (IL)-10, an anti-inflammatory cytokine, was reduced after CLI induction; whereas EPO, AG490, or combined EPO and AG490 treatments increased the expression levels of IL-10 in ischemic quadriceps with a synergistic pattern (Fig. 4c).

The mRNA expression levels of anti-oxidant genes, heme oxygenase (HO)-1, NAD(P)H:quinone oxidoreductase 1 (NQO1), glutathione reductase (GR), and glutathione peroxidase (GPx) were increased after CLI induction (Fig. 4d-g). Treatment with EPO, AG490, or combined EPO and AG490 all further increased the transcripts. Showing the same trend as IL-10, EPO and AG490 synergistically enhanced the expression levels of anti-oxidants (Fig. 4d-g).

### Phosphorylation of ERK1/2 with that activation of the JAK2/STAT pathway

**Fig. 5:**
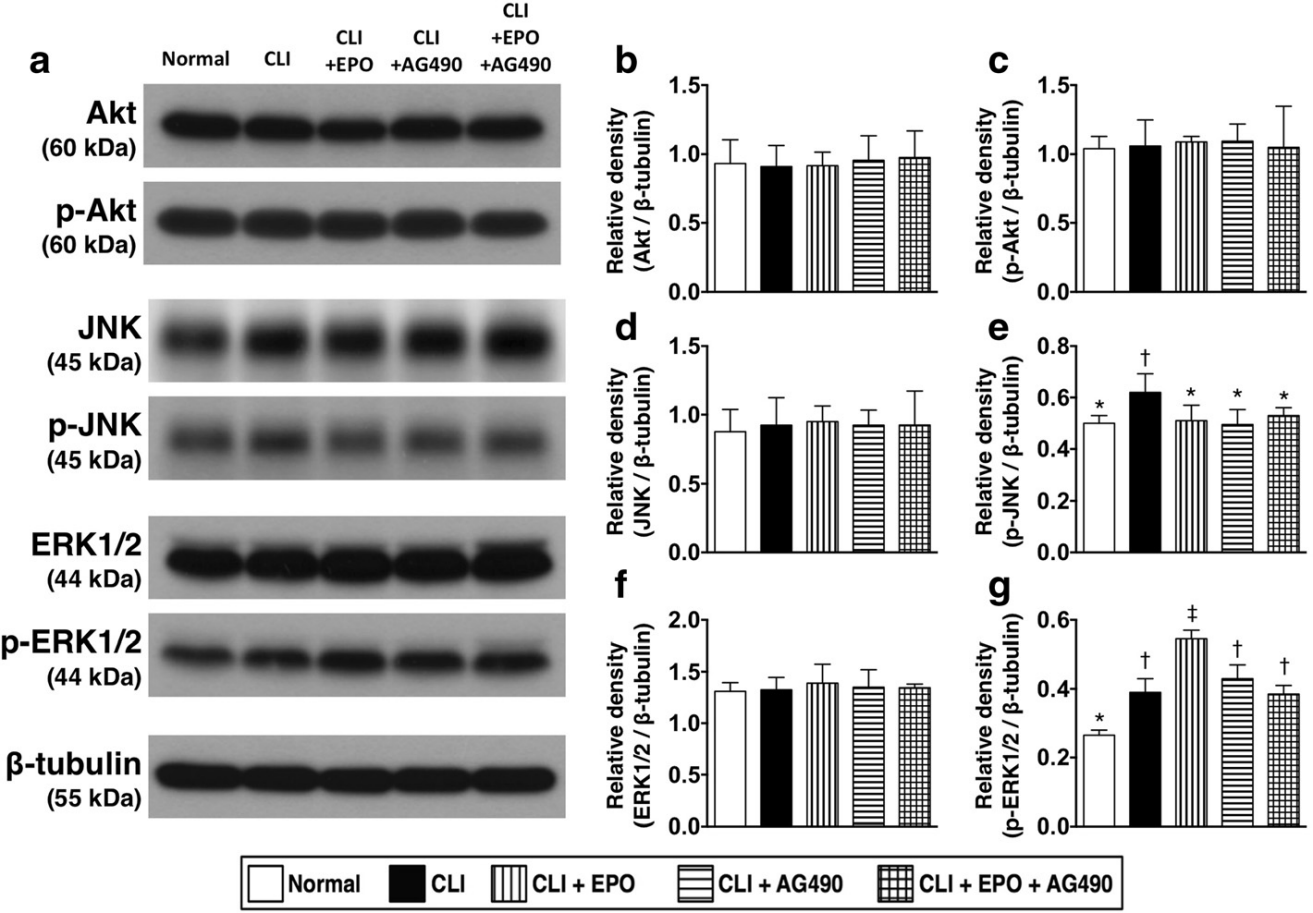
Expression and phosphorylation levels of Akt, JNK, and ERK1/2 in ischemic quadriceps. **a** Total and phosphorylated protein expression levels of Akt, JNK, and ERK1/2 were examined through Western blots with specific antibodies. **b** Expression levels of total Akt. **c** Expression levels of phosphorylated Akt. **d** Expression levels of total JNK. **e** Expression levels of phosphorylated JNK. **f** Expression levels of total ERK1/2. **g** Expression levels of phosphorylated ERK1/2. The protein expression levels are quantitated and normalized with the expression levels of β-tubulin. Statistical analysis used one-way ANOVA followed by Bonferroni multiple comparison post hoc test (*n* = 8 for each group). Symbols (*, †, ‡) indicate significance at p value less than 0.05

Although JAK2/STAT signaling is usually considered to be the downstream signal transduction of EPO, other kinase-based signals, including MEK/ERK and PI3K/Akt pathways have also been demonstrated to be involved in EPO-triggered intracellular signaling. Therefore, we next examined the total and phosphorylation levels of Akt, JNK, and ERR1/2 proteins in the ischemic quadriceps (Fig. 5a). Results showed that EPO, AG490, or combined EPO and AG490 treatment did not regulate the total expression level and phosphorylation levels of Akt after CLI induction (Fig. 5b and c). The total expression levels of JNK showed no difference among all groups (Fig. 5d), while the phosphorylation levels of JNK were increased by induction of CLI. However, EPO and AG490 did not regulate the activation of JNK (Fig. 5e) The total expression levels of ERK1/2 showed no difference among all groups (Fig. 5f), while the phosphorylation of ERK1/2 were increased after CLI induction and further enhanced with EPO treatment (Fig. 5g). However, AG490-only treatment or combined EPO and AG490 after CLI did not regulate the phosphorylation of ERK1/2.

### Activation of JAK2 signaling is important in EPO-mediated increased expression of EPO receptors

**Fig. 6:**
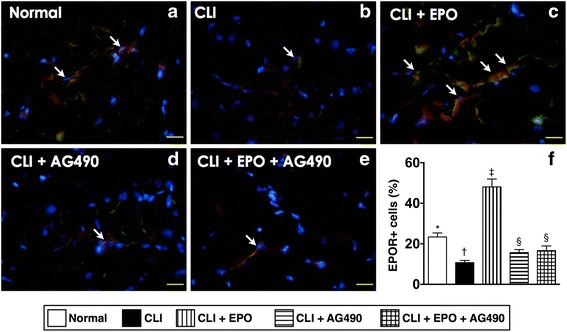
EPO administration increases the expression levels of EPO receptors in ischemic limbs. Twenty-four hours after CLI, quadriceps from ischemic limbs were isolated for cryosections and used for immunohistochemical staining to detect the expression and distribution of EPO receptor (EPOR). **a**-**e** The endothelial cells were recognized with antibodies against CD31 and shown in green color. The expression of EPOR was recognized with antibodies against EPOR and shown in red color. Arrows indicated the localization of EPOR positively stained (EPOR+) cells in quadriceps. **f** The quantitation of the number of EPOR+ cells. Statistical analysis used one-way ANOVA followed by Bonferroni multiple comparison post hoc test (*n* = 8 for each group). Symbols (*, †, ‡, §) indicate significance at p value less than .05

The expression levels of EPO receptors in endothelial cells were examined through immunofluorescence staining in quadriceps (Fig. 6). Results showed that the number of EPO positively stained (EPO+) endothelial cells was reduced after CLI induction, indicating the loss of endothelial cells in the acute phase after CLI induction (Fig. 6b). However, EPO treatment not only reverted the number of EPOR+ endothelial cells, but also increased the intensity of EPOR signals (Fig. 6c). Of importance, the increase of EPOR+ cells by EPO was blocked by additional AG490 treatment (Fig. 6e).

### Enhanced blood flow recovery with EPO and AG490 treatment

**Fig. 7:**
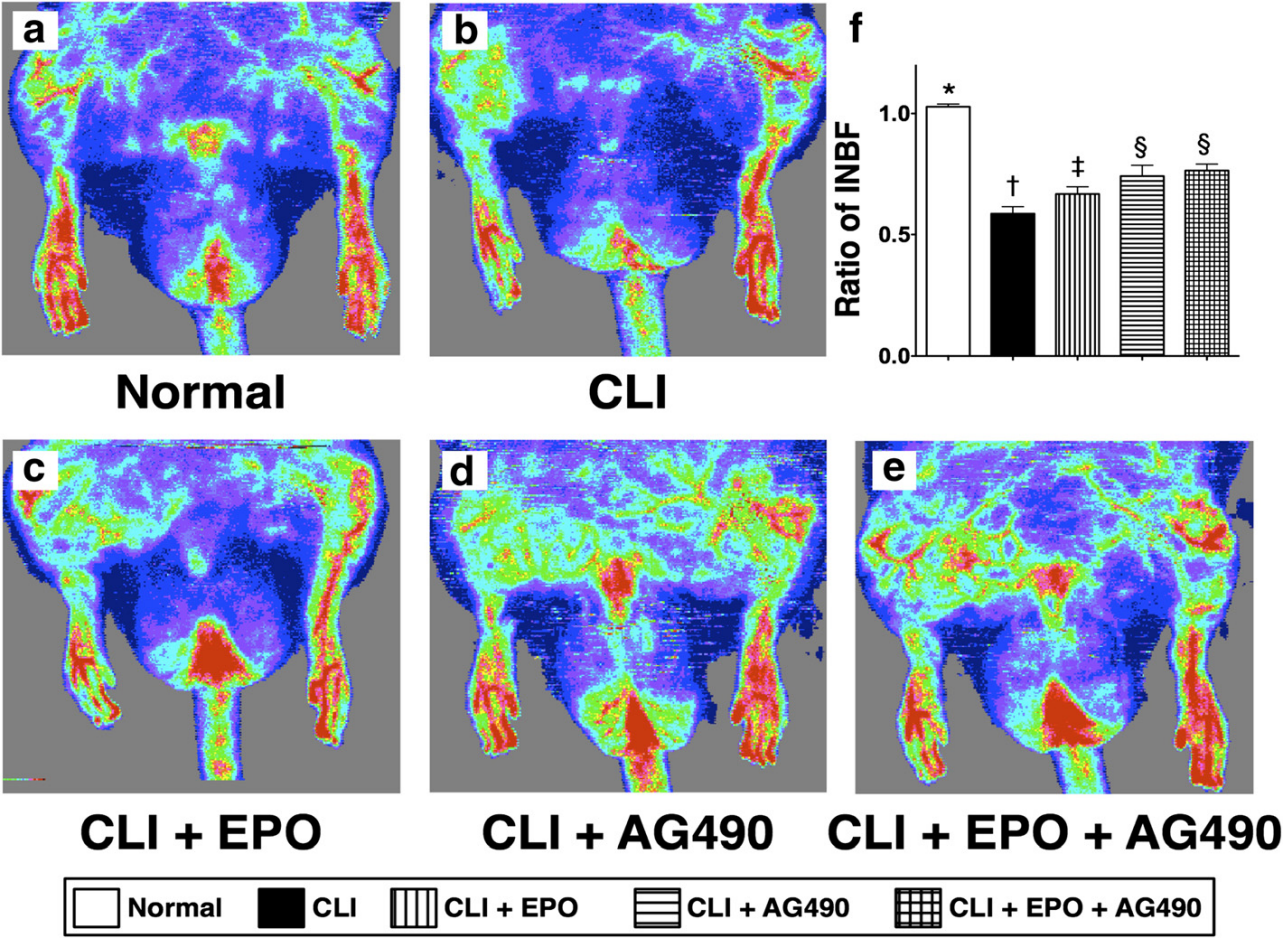
EPO and AG490 administrations increase the blood flow recovery after critical limb ischemia induction in rats. **a**-**e** Fourteen days after CLI, blood flows in the rat lower limbs were measured by laser Doppler. **f** Quantitation and calculation of blood flows. Statistical analysis using one-way ANOVA, followed by Bonferroni multiple comparison post hoc test (*n* = 8 for each group). Symbols (*, †, ‡) indicate significance at p value less than 0.05. INBF, ischemia to normal blood flow

**Fig. 8:**
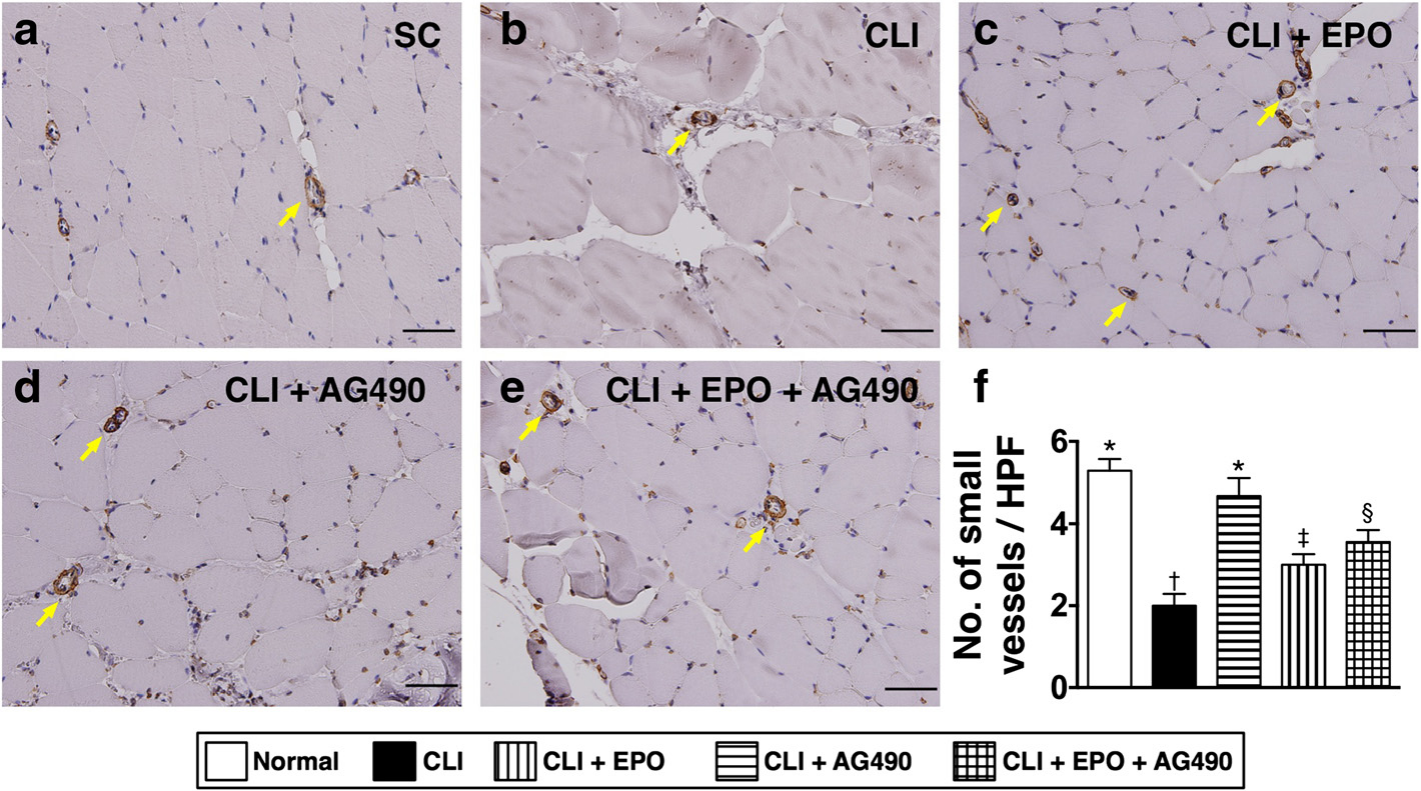
EPO and AG490 administrations increase the vessel density in quadriceps after critical limb ischemia induction in rats. The distribution of small vessels (diameter < 35 μm) was detected with antibodies against α-smooth muscle actin. **a** Quadricep section form normal rats. **b** Quadricep section form rats treated with critical limb ischemia (CLI). **c** Quadricep section form CLI rats treated with EPO. **d** Quadricep section form CLI rats treated with AG490. **e** Quadricep section form CLI rats treated with EPO and AG490. **f** Quantitation of number of small vessels in quadriceps. Yellow arrows indicate small vessels. Statistical analysis using one-way ANOVA, followed by Bonferroni multiple comparison post hoc test (*n* = 8 for each group). Symbols (*, †, ‡, §) indicate significance at p value less than 0.05

Fourteen days after CLI induction, we applied laser Doppler analysis to examine the blood flow recovery in the ischemic limbs. After quantitation, the ratio of ischemia to normal blood flow (INBF) was used as the parameter to determine the condition of blood flow recovery (Fig. 7). The ratio of INBF in the CLI only group was significantly lower than in the normal group by day 14 after CLI. It is interesting that not only EPO, but also AG490 enhanced the blood flow recovery after CLI. However, combined EPO and AG490 treatment did not show synergistic effects in enhancing blood flow recovery in ischemic limbs. To further confirm the enhanced blood flow recovery by EPO and AG490, immunohistochemical staining against α-smooth muscle actin (α-SMA) was performed to determine the number of vessels in ischemic limbs (Fig. 8). As the result of laser Doppler examination, the numbers of small vessels (diameter <35 μm) in the quadriceps was reduced by CLI induction and reverted with EPO treatment. AG490-only treatment also increased the number of small vessels; however, the increment was fewer than that in those treated with EPO. No synergistic effect was found in CLI rats receiving EPO and AG490 combined therapy.

## Discussion

In the present study, we directly applied an experimental rat model with critical limb ischemia to evaluate the effects of EPO and JAK2 inhibitor AG490 in preventing apoptosis as well as in enhancing blood recovery in ischemic limbs. In addition, we also performed biochemical analysis, histopathological examination, and immunostaining to detect the activation of underlying signaling responding to CLI insults and EPO treatment. The results suggest that EPO can prevent cellular apoptosis and reduce the size of the infarct area in the ischemic quadriceps during the acute phase of CLI (Figs. 2 and 3). We also found that the phosphorylation of STAT1 and STAT5, but not STAT3, was activated by EPO-mediated JAK2 activation in the CLI areas (Fig. 1). Moreover, our results showed that the JAK2 inhibitor AG490 blocked the activation of JAK2/STAT signaling and abolished the anti-apoptotic efficacy of EPO (Figs. 1-3). However, AG490-only treatment increased the synthesis of anti-oxidants and also reduced the gene expressions of inflammatory factors (Fig. 4). It is worth noting that the combined EPO and AG490 treatment showed synergistic effects in enhancing anti-oxidation and anti-inflammation. At day 14 after CLI, we found that EPO, AG490 and combined EPO and AG490 therapies all increased the blood flow recovery after CLI (Figs. 7 and 8). In addition, results from Western blots showed that the activation of JNK and ERK pathways in quadriceps were regulated by ischemic stress, EPO, and AG490 (Fig. 5).

### EPO-mediated intracellular signaling in ischemic limbs

Among STAT transcription factors, STAT5 is the most prominent substrate to be phosphorylated with JAK2 [39, 40], while phosphorylated STAT3 and STAT1 are only found in certain kinds of cells [41, 42]. In the present study, we noted that the phosphorylation of STAT5 and STAT1, but not STAT3, were upregulated by EPO and along with the phosphorylation levels of JAK2. In addition to the JAK2/STAT pathways, several mitogen-activated protein kinases (MAPKs) signaling cascades, including ERK, p38, and JNK, have been reported to play roles in delivering ERO/EPOR signals [43–45]. Results in this study also demonstrated that the expression levels and phosphorylation levels of ERK1/2 were upregulated by EPO and reverted with additional AG490 treatment (Fig. 5). Although previous studies considered the activation of PI3K is important for EPO-mediated phosphorylation of ERK [46], the activation of Akt, another PI3K kinase substrate, did not increase after EPO treatment in ischemic quadriceps (Fig. 5). Hence, instead of PI3K, other kinase facility, i.e. Ras/Raf, may contribute in the EPO-mediated, JAK2-dependent, activation of MAPKs after CLI. Although tyrosine phosphorylation of STATs with JAK kinase is found in most cells, some reports demonstrated the existence of JAK2-independent phosphorylation of STAT5 [47, 48]. To clarify this issue, we combined EPO and AG490 to treat rat CLI and found that STAT5 activation in ischemic quadriceps was in a JAK2-dependent manner. The inhibitory effect of AG490 in JAK2/STAT signaling has been found in tissues with different pathological stresses, including myocardial hypertrophy [49], liver ischemia-reperfusion [35], traumatic brain injury [50], and chronic renal disease [51]. In the present study, we further confirmed the effect of AG490 in inhibiting the activation and JAK2 and its downstream phosphorylation of STAT1, STAT3, and STAT5 in the critical limb ischemia model.

### Upregulation of anti-inflammatory and anti-oxidative genes by EPO and AG490

In addition to roles in erythropoiesis, other functions of EPO, including anti-apoptosis and angiogenesis, have also been demonstrated [22, 30]. In this study, through the CLI rat model, we found that EPO regulated the gene expression levels of inflammatory and anti-oxidative proteins and showed a protective effect against inflammation and oxidation. Of note, AG490 also provided similar anti-inflammatory and anti-oxidation effects, despite its roles in blockage of JAK2 activity and impairing anti-apoptosis. The distinct role of AG490 may be through inhibition of JAK2, which reduces gene expression of anti-apoptotic factors in endothelial and skeletal muscle cells, and also inhibits inflammatory cytokine release, proliferation, and activation in inflammatory cells. Hence, the distinct physiological roles of AG490 on different kinds of cells may provide contradictory effects in enhancing recovery from ischemic injury.

### Combined EPO and AG490 to treat CLI

Although the anti-inflammatory capacity of AG490 reduced the generation of inflammatory factors and also increased the expression levels of anti-oxidants, its role in blocking JAK2 activity impaired the anti-apoptotic effects of EPO in the acute phase of CLI. These seemingly contradictory roles may explain the controversy surrounding the effects of AG490 in the treatment of ischemia-reperfusion injury in different organs [35, 52–54]. In this study, the examination of blood flow examination using laser Doppler analysis also showed both EPO and AG490 treatments enhance the blood flow recovery after CLI; however, no additional benefit was found in the combined treatment group. Since JAK2 activity is critical in the function of EPO in preventing ischemia-induced cellular apoptosis, the application of AG490 should be avoided in the acute phase of CLI. Combined EPO and delayed AG490 adminstration may provide better therapeutic efficacy in treating CLI.

## Conclusion

This study indicates that JAK2-dependent STAT activation plays an important role in EPO-mediated enhanced blood flow recovery after CLI induction in rats. Although AG490 inhibits the EPO-induced JAK2/STAT activation in the acute phase of CLI, the benefits of AG490 in anti-inflammation and anti-oxidation still provide a better outcome in rats with CLI. The functional effects of EPO in anti-apoptosis, anti-inflammation, and anti-oxidation provide therapeutic efficacy for injury recovery after critical limb ischemia.
